# Supplementary material for: Nasopharyngeal lymphatic plexus is a hub for cerebrospinal fluid drainage
Source: Nature. 2024 Jan 10;625(7996):768–77. doi: 10.1038/s41586-023-06899-4 (PMC10808075; doi:10.1038/s41586-023-06899-4)
Supplement: Supplementary file 1 — Supplementary Figs. 1–12. [file 41586_2023_6899_MOESM1_ESM.pdf]

nature portfolio

https://doi.org/10.1038/s41586-023-06899-4

Supplementary information

Nasopharyngeal lymphatic plexus is a hub for cerebrospinal fluid drainage

In the format provided by the authors and unedited

Supplementary Information

Nasopharyngeal lymphatic plexus is a hub for cerebrospinal fluid drainage

Jin-Hui Yoon[1,#], Hokyung Jin[1,2,#], Hae Jin Kim[3,#], Seon Pyo Hong[1], Myung Jin Yang[1,2], Ji Hoon Ahn[1], Young-Chan Kim[1,2], Jincheol Seo[4], Yongjeon Lee[4], Donald M. McDonald[5], Michael J. Davis[3,*], Gou Young Koh[1,2,*]

[1]Center for Vascular Research, Institute for Basic Science, Daejeon 34141, Republic of Korea
[2]Graduate School of Medical Science and Engineering, Korea Advanced Institute of Science and Technology (KAIST), Daejeon 34141, Republic of Korea
[3]Department of Medical Pharmacology and Physiology, University of Missouri, Columbia, Missouri 65212, United States of America
[4]National Primates Research Center, Korea Research Institute of Bioscience and Biotechnology, Cheongju 28116, Republic of Korea
[5]Department of Anatomy, Cardiovascular Research Institute, and Helen Diller Family Comprehensive Cancer Center, University of California, San Francisco, San Francisco, California 94143-0452, United States of America

[#]These authors contributed equally.
*These authors jointly supervised this work.

Correspondence: Gou Young Koh (gykoh@kaist.ac.kr)
Michael J. Davis (davismj@health.missouri.edu)

Contents

Supplementary Figures 1-12

Supplementary Figure 1. Distinctive features of the nasopharyngeal lymphatic plexus

Supplementary Figure 2. TMR-Dextran outflow through nasopharyngeal lymphatic plexus after intracisternal infusion in Prox1-GFP mice

Supplementary Figure 3. Similar distributions of TMR-Dextran in the superficial and deep cervical lymph nodes after the intracisternal infusion

Supplementary Figure 4. Outflow of 70 kD TMR-Dextran, TR-ovalbumin, or FluoSpheres through nasopharyngeal lymphatic plexus after intracisternal infusion

Supplementary Figure 5. Morphological landscape of lymphatic connections from the nasopharyngeal lymphatic plexus to deep cervical lymph nodes in Prox1-GFP mice

Supplementary Figure 6. Distribution of tyrosine phosphatase immunoreactive adrenergic neurons along deep cervical lymphatics

Supplementary Figure 7. Connections of basolateral meningeal lymphatics to dorsal meningeal lymphatics and to lateral deep cervical lymphatics through jugular foramen

Supplementary Figure 8. Preferential distribution of TMR-Dextran in the anterior and middle regions of the skull base after the intracisternal infusion

Supplementary Figure 9. Timing of TMR-Dextran clearance to deep cervical lymph nodes after intracisternal or intrahippocampal infusion in mice

Supplementary Figure 10. Ageing-related alterations in nasopharyngeal lymphatic plexus

Supplementary Figure 11. Similar size and lymphatic area of deep cervical lymph nodes in adult and aged mice

Supplementary Figure 12. Heterogeneous expression of Ptx3 in lymphatic endothelial cells of the nasopharyngeal lymphatic plexus

Supplementary Videos 1-6

Supplementary video 1. Three-dimensional reconstruction of $Prox1^+$ nasopharyngeal lymphatic plexus of Prox1-GFP mouse

Supplementary Video 2. Intravital imaging for TMR-Dextran outflow through the nasopharyngeal lymphatic plexus at 30 min after intracisternal infusion of TMR-Dextran into Prox1-GFP mouse

Supplementary Video 3. Intravital imaging for TMR-Dextran outflow through the nasopharyngeal lymphatic plexus at onset and 120 min after intracisternal infusion of TMR-Dextran into Prox1-GFP mouse

Supplementary Video 4. Light-sheet fluorescence microscopic image showing $Prox1^+$/$LYVE1^+$ lymphatics near the $Prox1^+$ pituitary gland area that extends to the nasopharyngeal lymphatic plexus along cranial nerve V and cavernous sinus in Prox1-GFP mouse

Supplementary Video 5. Light-sheet fluorescence microscopic image showing the lymphatics around the pterygopalatine artery that connect to the posterior nasal lymphatic plexus and nasopharyngeal lymphatic plexus in Prox1-GFP mouse

Supplementary video 6. Three-dimensional reconstruction of $Prox1^+$ connecting lymphatics between dural lymphatics and olfactory lymphatics in Prox1-GFP mouse

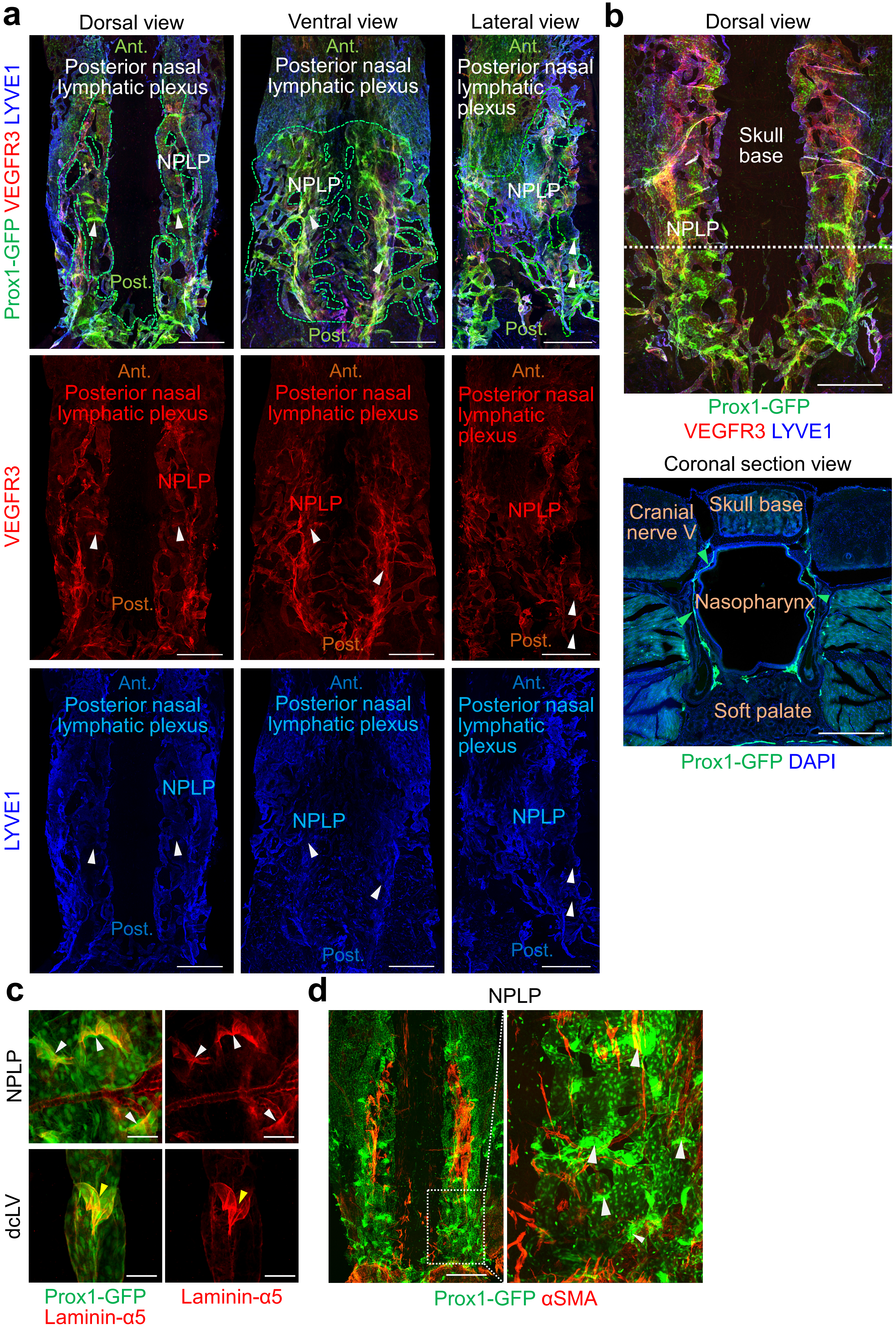
a
Dorsal view
Ventral view
Lateral view
Prox1-GFP VEGFR3 LYVE1
Ant.
Posterior nasal lymphatic plexus
NPLP
Post.
VEGFR3
LYVE1
b
Dorsal view
Skull base
NPLP
Prox1-GFP
VEGFR3 LYVE1
Coronal section view
Cranial nerve V
Skull base
Nasopharynx
Soft palate
Prox1-GFP DAPI
c
NPLP
dcLV
Prox1-GFP
Laminin-α5
Laminin-α5
d
NPLP
Prox1-GFP αSMA

Supplementary Fig. 1. Distinctive features of the nasopharyngeal lymphatic plexus
a, Immunofluorescence images of three views of whole mounts of the nasopharyngeal lymphatic plexus (NPLP) and posterior nasal lymphatic plexus of Prox1-GFP (green) mice after staining for VEGFR3 (red) and LYVE1 (blue). NPLP lymphatics are outlined by green dashed lines. White arrowheads mark lymphatic valves with strong Prox1 staining. Scale bars, 500 μm. Similar findings were obtained from n = 4 mice in three independent experiments. Ant., anterior; Post., posterior anatomical position.
b, Immunofluorescence images of whole mount (upper) and coronal section (lower) of the NPLP. White dashed line in upper panel marks the plane of section of the lower panel. Green arrowheads mark the border of the NPLP. Scale bars, 500 μm. Similar findings were obtained from n = 4 mice in three independent experiments.
c, Immunofluorescence images of whole mounts comparing unusual irregular and linear valves (white arrowheads) in the NPLP and usual semilunar valves (yellow arrowheads) in deep cervical lymphatics (dcLV) in Prox1-GFP mice. Both types of valve have strong staining for Prox1 and laminin-α5. Scale bars, 200 μm. Similar findings were obtained from n = 4 mice in two independent experiments.
d, Immunofluorescence images of whole mount showing absence of αSMA^+ smooth muscle cells around $Prox1^+$ NPLP (left panel). White dashed-line boxed region of NPLP is enlarged in the right panel. White arrowheads mark lymphatic valves with strong Prox1 staining. Scale bar, 500 μm. Similar findings were obtained from n = 4 mice in two independent experiments.

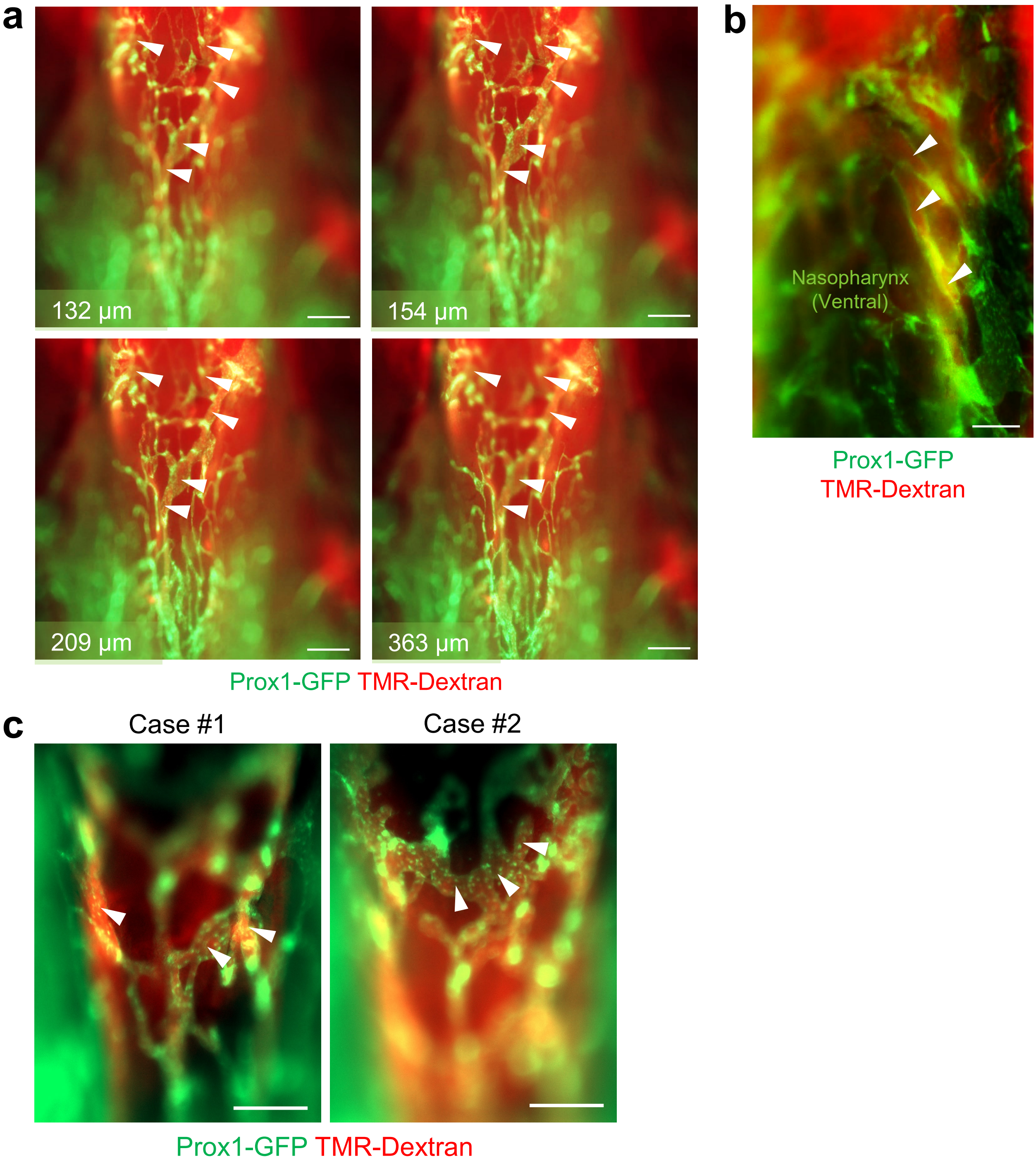

Supplementary Fig 2. TMR-Dextran outflow through nasopharyngeal lymphatic plexus after intracisternal infusion in Prox1-GFP mice

a, Focal plane sequence of fluorescence images showing TMR-Dextran outflow through the nasopharyngeal lymphatic plexus (NPLP) at 30 min after intracisternal infusion (see also **Fig. 2d** and **Supplementary Video 2**). TMR-Dextran fluorescence (red) is strong in (white arrowheads) and around the Prox1$^+$ lymphatic plexus. Values in lower left show focal plane distance (µm) from the skull base. Scale bars, 500 µm. Similar findings were obtained from n = 5-6 mice in three independent experiments.

b, Fluorescence image showing TMR-Dextran outflow in the ventral region of the NPLP at 30 min after intracisternal infusion. TMR-Dextran fluorescence (red) is strong in (white arrowheads) and around the Prox1$^+$ lymphatic plexus. Scale bar, 200 µm. Similar findings were obtained from n = 4 mice in two independent experiments.

c, Fluorescence images showing two examples (Case #1 and #2) that demonstrate TMR-Dextran outflow through the NPLP at 30 min after the intracisternal infusion without exsanguination at the end of the experiment. This control experiment shows strong TMR-Dextran fluorescence (red) in (white arrowheads) the Prox1$^+$ NPLP. Scale bars, 200 µm. Similar findings were obtained from n = 4 mice in two independent experiments.

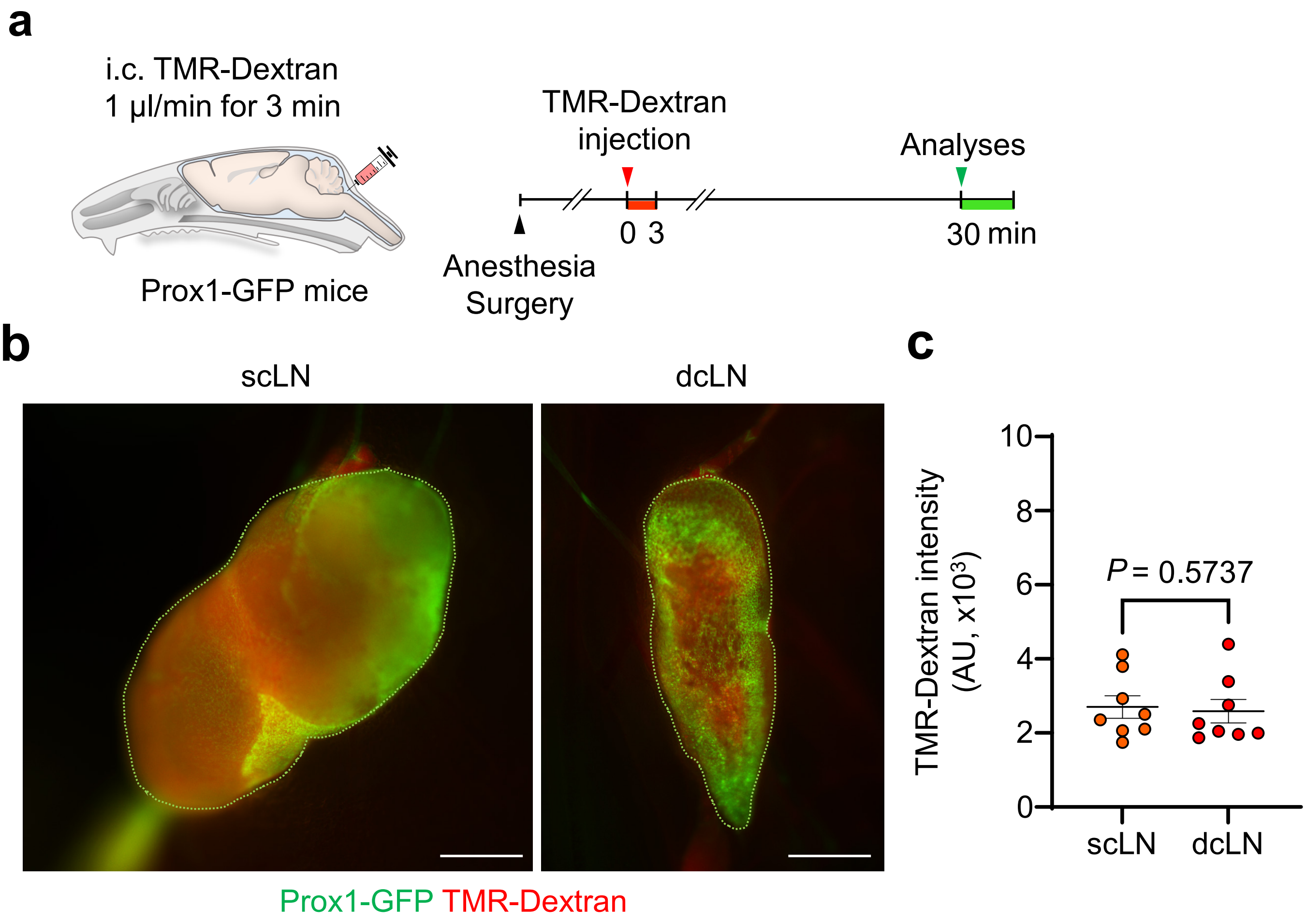

Supplementary Fig. 3. Similar distributions of TMR-Dextran in the superficial and deep cervical lymph nodes after the intracisternal infusion

a, Diagram of the experimental sequence of intracisternal (i.c.) infusion of TMR-Dextran at 1.0 µl/min for 3 min into Prox1-GFP mice followed by measurement of TMR-Dextran fluorescence in the superficial (scLN) and deep (dcLN) cervical lymph nodes at 30 min.

b, c, Images comparing strong but similar TMR-Dextran fluorescence (red) both in scLN and (outlined by green dashed lines) 30 min after i.c. infusion. Scale bars, 500 µm. Two values (from right and left lymph nodes) were obtained from one mouse and n=4 mice per group in four independent experiments. Dots and bars present mean ± s.e.m. AU, arbitrary unit. *P* value was calculated by two-tailed Mann-Whitney test.

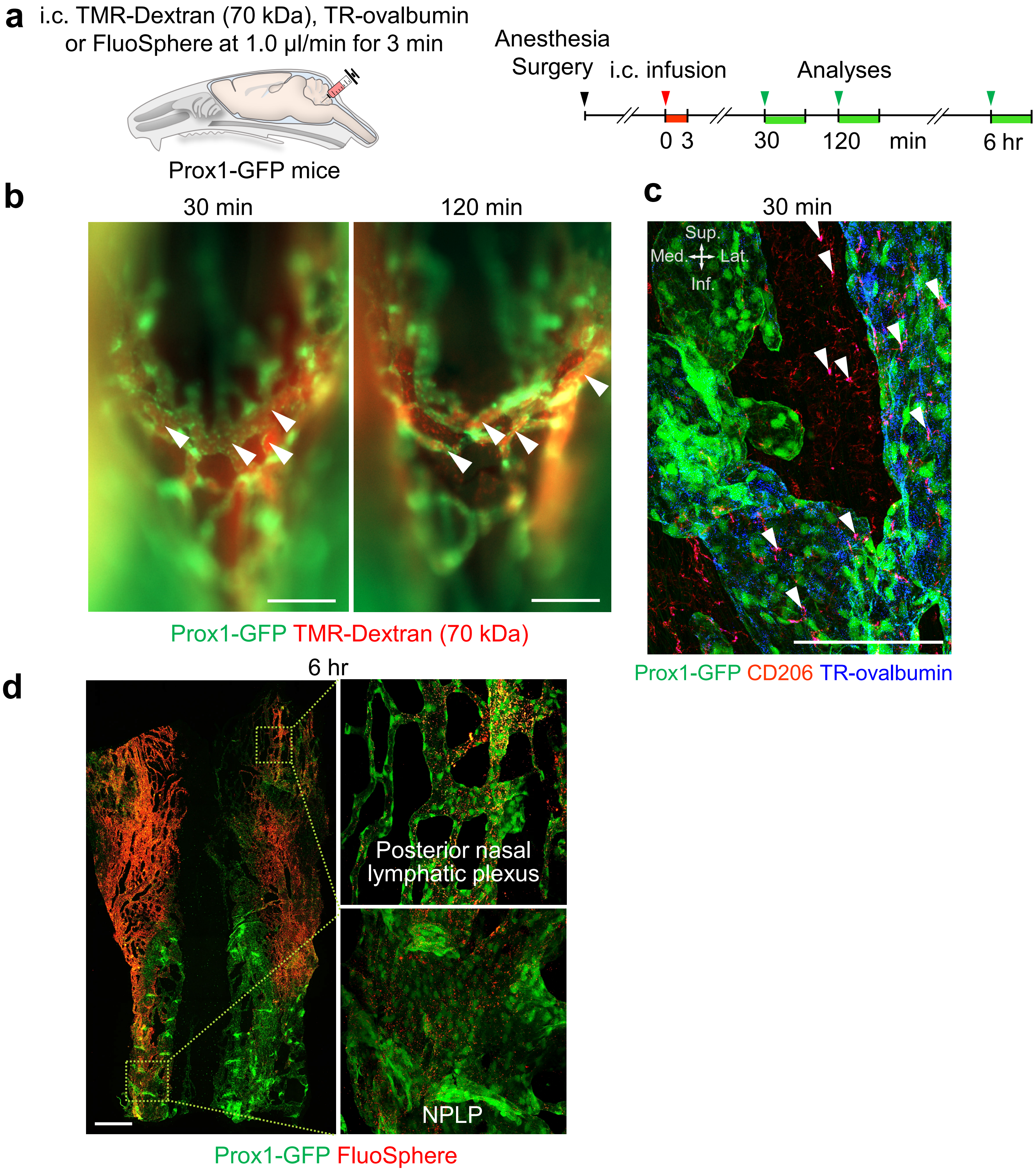

Supplementary Fig 4. Outflow of 70 kD TMR-Dextran, TR-ovalbumin, or FluoSpheres through nasopharyngeal lymphatic plexus after intracisternal infusion

a, Diagram showing the site of intracisternal (i.c.) infusion of TMR-Dextran (70 kDa), Texas Red (TR)-ovalbumin, or FluoSpheres at 1.0 µl/min into Prox1-GFP mice and sequence for obtaining intravital images at 30 and 120 min, and 6 hr after the infusion.

b-d, Fluorescence images showing 70 kDa TMR-Dextran (white arrowheads in **b**) within lymphatics of the nasopharynx and TR-ovalbumin in CD206-immunoreactive macrophages (white arrowheads in **c**) around the nasopharyngeal lymphatic plexus (NPLP) at 30 and 120 min after infusion. **d,** FluoSpheres within the posterior nasal and nasopharyngeal lymphatic plexus at 6 hr after infusion. Scale bars, 200 µm. Anatomical positions are indicated in the upper left corner in **c**. Similar findings were obtained from n = 4 mice in two independent experiments for each infusion experiment. Sup., superior; Inf., inferior; Med., medial; Lat., lateral anatomical position.

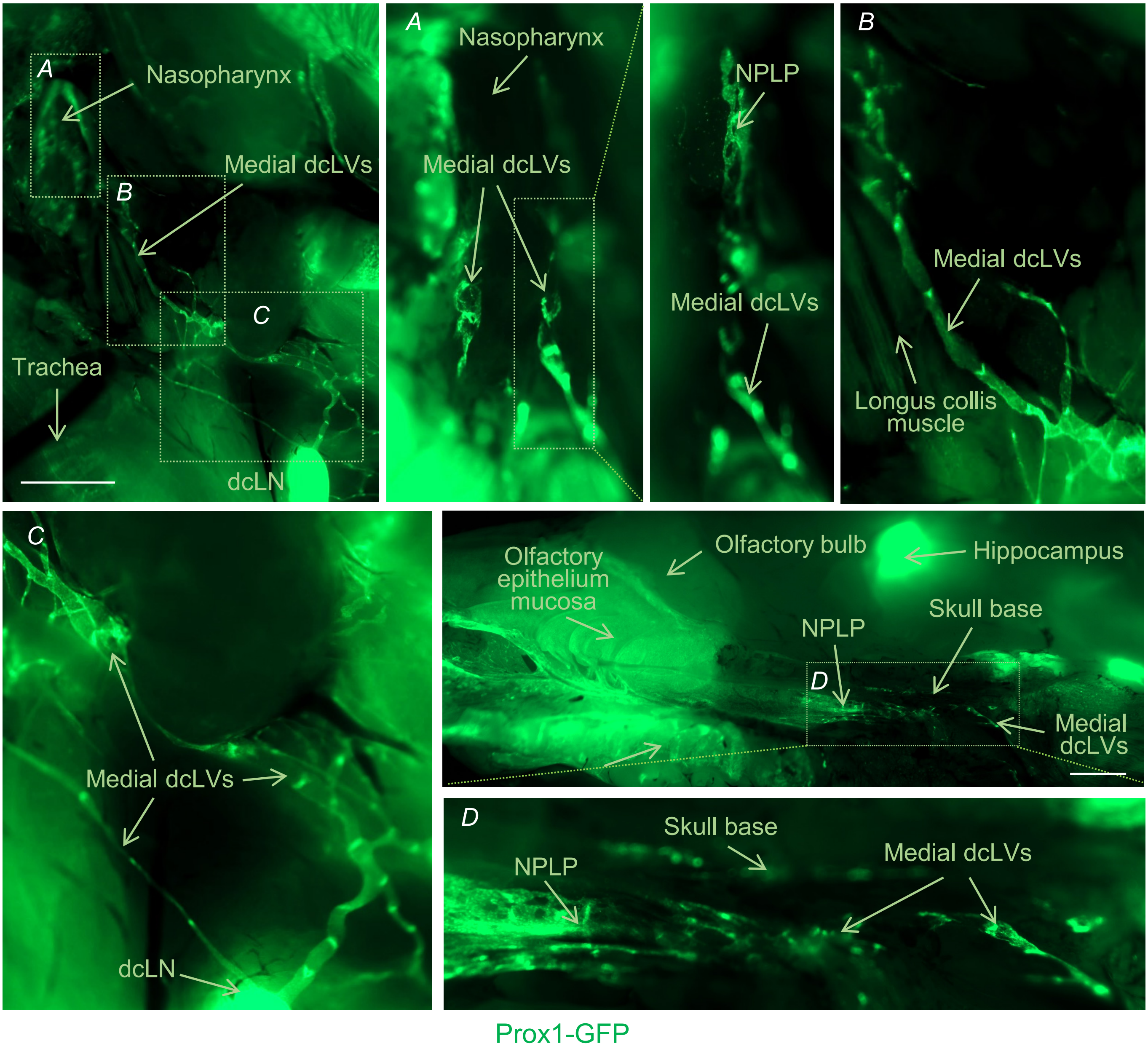

Supplementary Fig. 5. Morphological landscape of lymphatic connections from the nasopharyngeal lymphatic plexus to deep cervical lymph nodes in Prox1-GFP mice
Fluorescence stereomicroscopic images of the nasopharyngeal lymphatic plexus (NPLP), medial deep cervical lymphatics (medial dcLVs), and deep cervical lymph nodes (dcLN) in the skull base, neck, and olfactory bulb/mucosa regions of Prox1-GFP mice after postmortem dissection. Scale bars, 4 mm. Similar findings were obtained from n = 6 mice in three independent experiments.

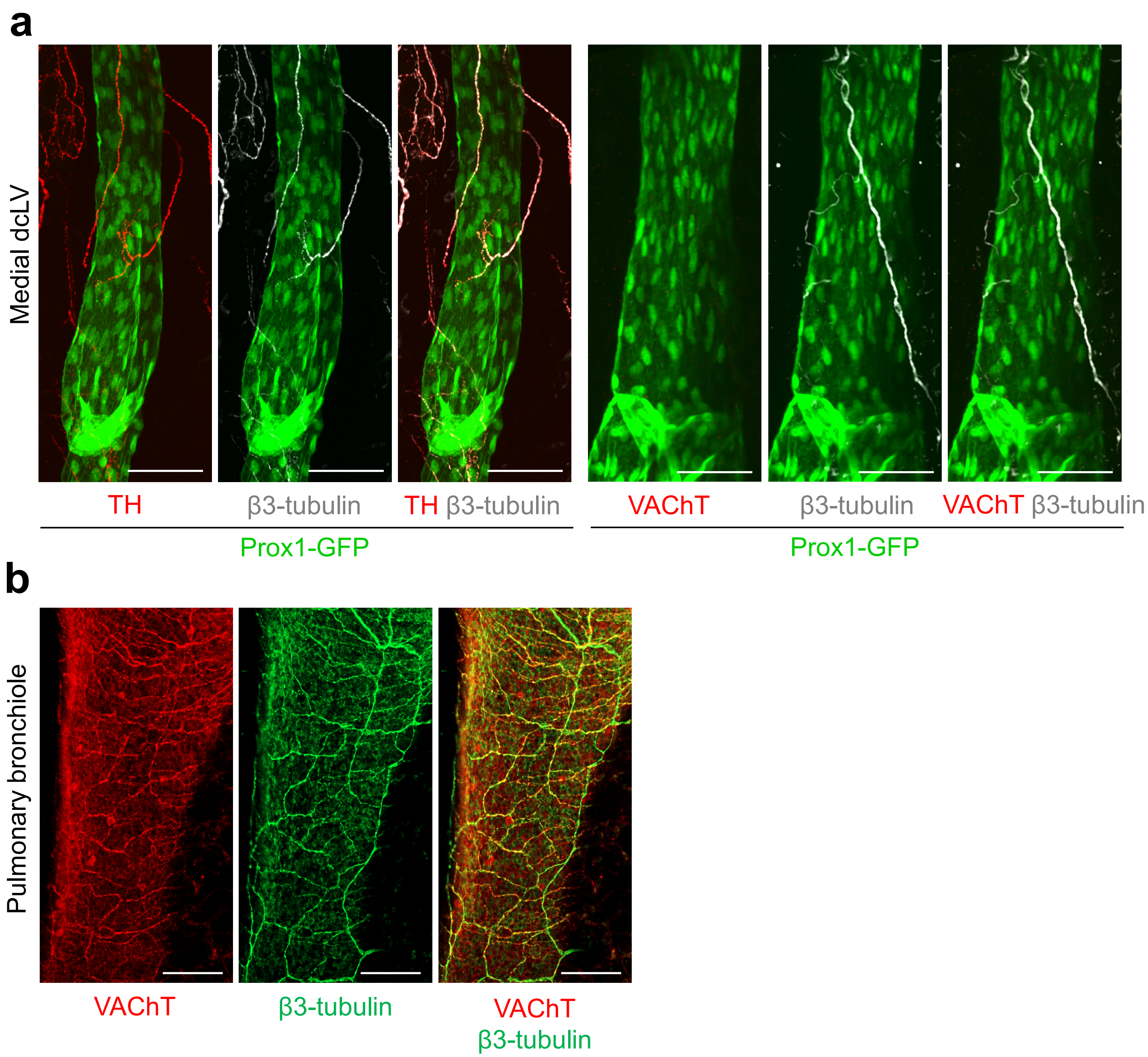

Supplementary Fig. 6. Distribution of tyrosine phosphatase immunoreactive adrenergic neurons along deep cervical lymphatics

a,**b**, Immunofluorescence images of whole mounts showing the abundance and distribution adrenergic axons with tyrosine phosphatase (TH)$^{+}$/β-tubulin immunoreactivities and cholinergic axons with vesicular acetylcholine transporter (VAChT)$^{+}$/β-tubulin immunoreactivities along a deep cervical lymphatic and a pulmonary bronchiole. Adrenergic axons are sparse but predominate along the deep cervical lymphatics. By comparison, axons are abundant on the bronchiole but cholinergic axons predominate. Scale bars, 100 μm. Similar findings were obtained from n = 3 mice in two independent experiments.

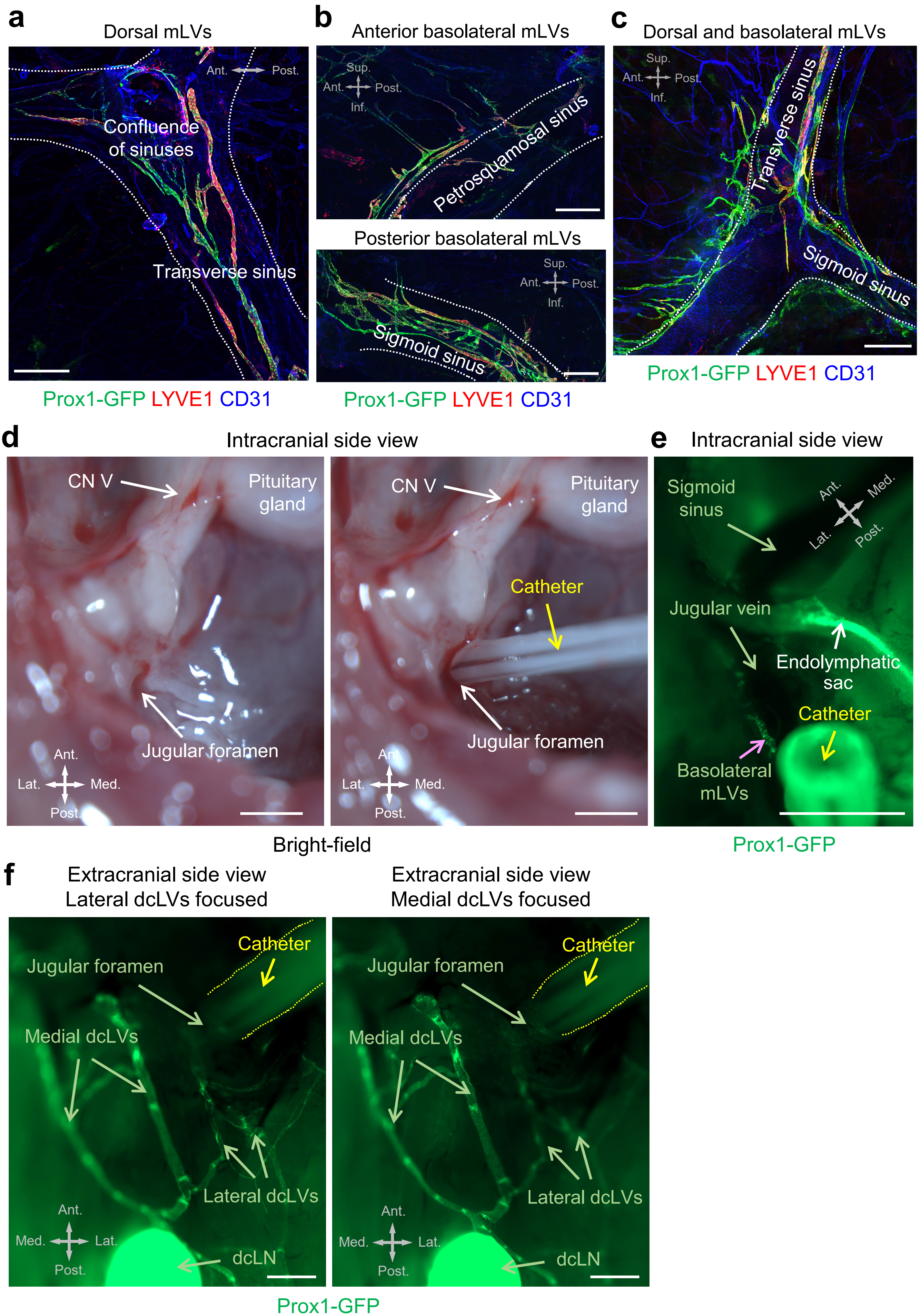

a
Dorsal mLVs
Ant. Post.
Confluence of sinuses
Transverse sinus
Prox1-GFP LYVE1 CD31
b
Anterior basolateral mLVs
Sup.
Ant. Post.
Inf.
Petrosquamosal sinus
Posterior basolateral mLVs
Sup.
Ant. Post.
Inf.
Sigmoid sinus
Prox1-GFP LYVE1 CD31
c
Dorsal and basolateral mLVs
Sup.
Ant. Post.
Inf.
Transverse sinus
Sigmoid sinus
Prox1-GFP LYVE1 CD31
d
Intracranial side view
CN V
Pituitary gland
Jugular foramen
Ant.
Lat. Med.
Post.
CN V
Pituitary gland
Catheter
Jugular foramen
Ant.
Lat. Med.
Post.
Bright-field
e
Intracranial side view
Sigmoid sinus
Ant. Med.
Lat. Post.
Jugular vein
Endolymphatic sac
Catheter
Basolateral mLVs
Prox1-GFP
f
Extracranial side view
Lateral dcLVs focused
Catheter
Jugular foramen
Medial dcLVs
Lateral dcLVs
Ant.
Med. Lat.
Post.
dcLN
Extracranial side view
Medial dcLVs focused
Catheter
Jugular foramen
Medial dcLVs
Lateral dcLVs
Ant.
Med. Lat.
Post.
dcLN
Prox1-GFP

Supplementary Fig. 7. Connections of basolateral meningeal lymphatics to dorsal meningeal lymphatics and to lateral deep cervical lymphatics through jugular foramen

a-c, Immunofluorescence images of whole mounts showing connections of dorsal and basolateral meningeal lymphatics (mLVs) around the transverse sinus, petrosquamosal sinus, and sigmoid sinus of Prox1-GFP mice. Anatomical positions are indicated in the upper corners. Scale bars, 500 µm. Similar findings were obtained from n = 3 mice in two independent experiments. Ant., anterior; Post., posterior; Sup., superior; Inf., inferior anatomical position.

d,e, Bright-field and fluorescence images of the skull base of Prox1-GFP mice. Jugular foramen is marked by a catheter (24 Gauge, fluorinated ethylene propylene). Prox1+ GFP basolateral mLVs (pink arrow) pass through the jugular foramen. Anatomical positions are indicated in the lower left or upper right corner. Scale bars, 1 mm. Similar findings were obtained from n = 4 mice in four independent experiments. Ant., anterior; Post., posterior; Lat., lateral; Med., medial anatomical position.

f, Fluorescence images showing the distribution of lymphatics in the head and neck of Prox1-GFP mice. Jugular foramen is marked by a catheter (24 Gauge, fluorinated ethylene propylene). Lateral deep cervical lymphatics (dcLVs) pass through the jugular foramen and connect to the deep cervical lymph node (dcLN). Anatomical positions are indicated in the lower left corner. Scale bars, 500 µm. Similar findings were obtained from n = 4 mice in four independent experiments. Ant., anterior; Post., posterior; Lat., lateral; Med., medial anatomical position.

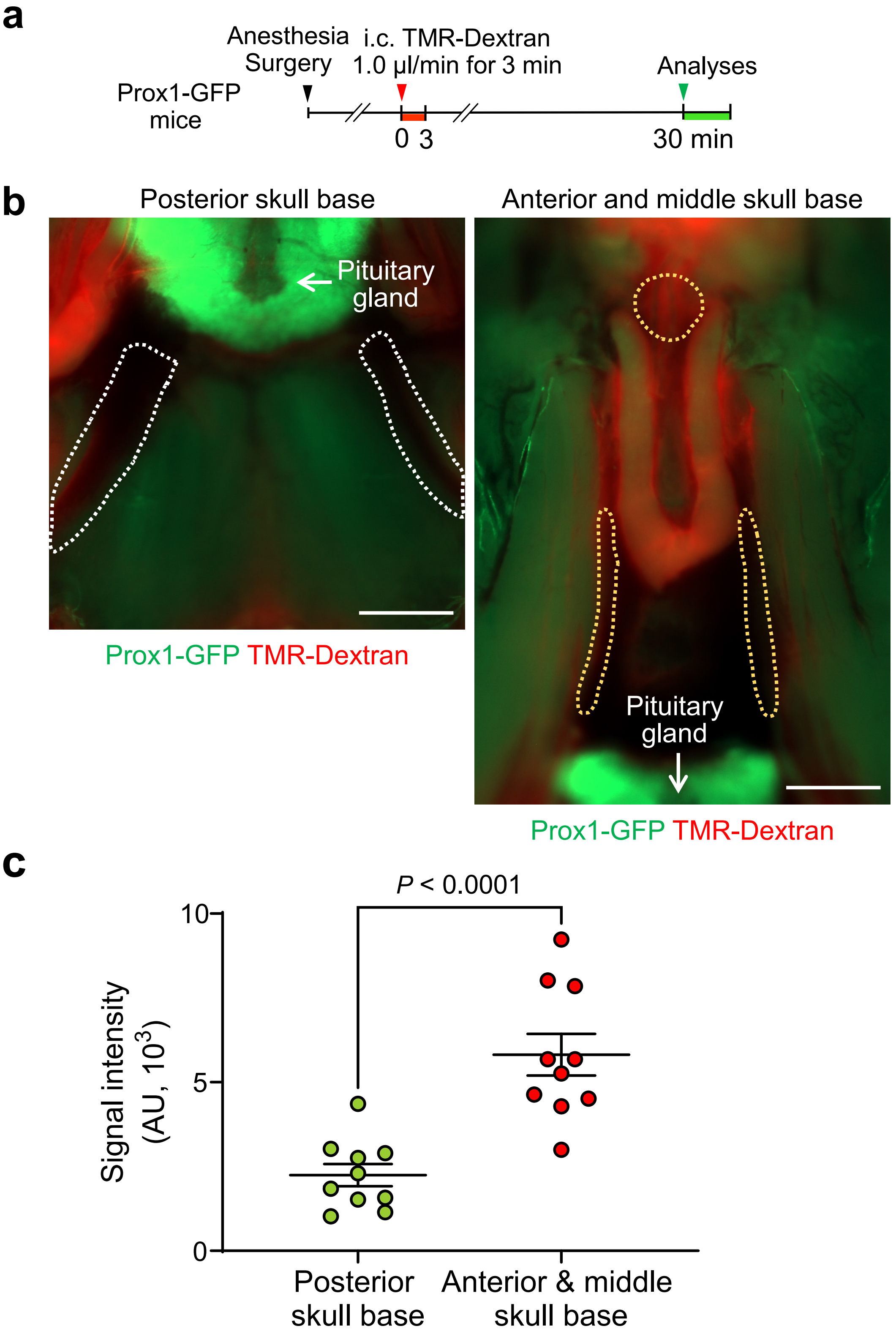

Supplementary Fig. 8. Preferential distribution of TMR-Dextran in the anterior and middle regions of the skull base after the intracisternal infusion

a, Diagram of the experimental sequence of intracisternal (i.c.) infusion of TMR-Dextran at 1.0 μl/min for 3 min into Prox1-GFP mice followed by measurement of TMR-Dextran fluorescence in the anterior/middle and posterior regions of the skull base at 30 min.

b, c, Images comparing the weak TMR-Dextran fluorescence (red) in the posterior region (outlined by white dashed lines) with the strong fluorescence in anterior and middle regions (outlined by yellow dashed lines) at the skull base 30 min after i.c. infusion. Scale bars, 1 mm. Each dot is the value for one mouse; n = 10 mice/group from three independent experiments. Error bars indicate mean ± s.e.m. *P* value was calculated by two-tailed Mann-Whitney test.

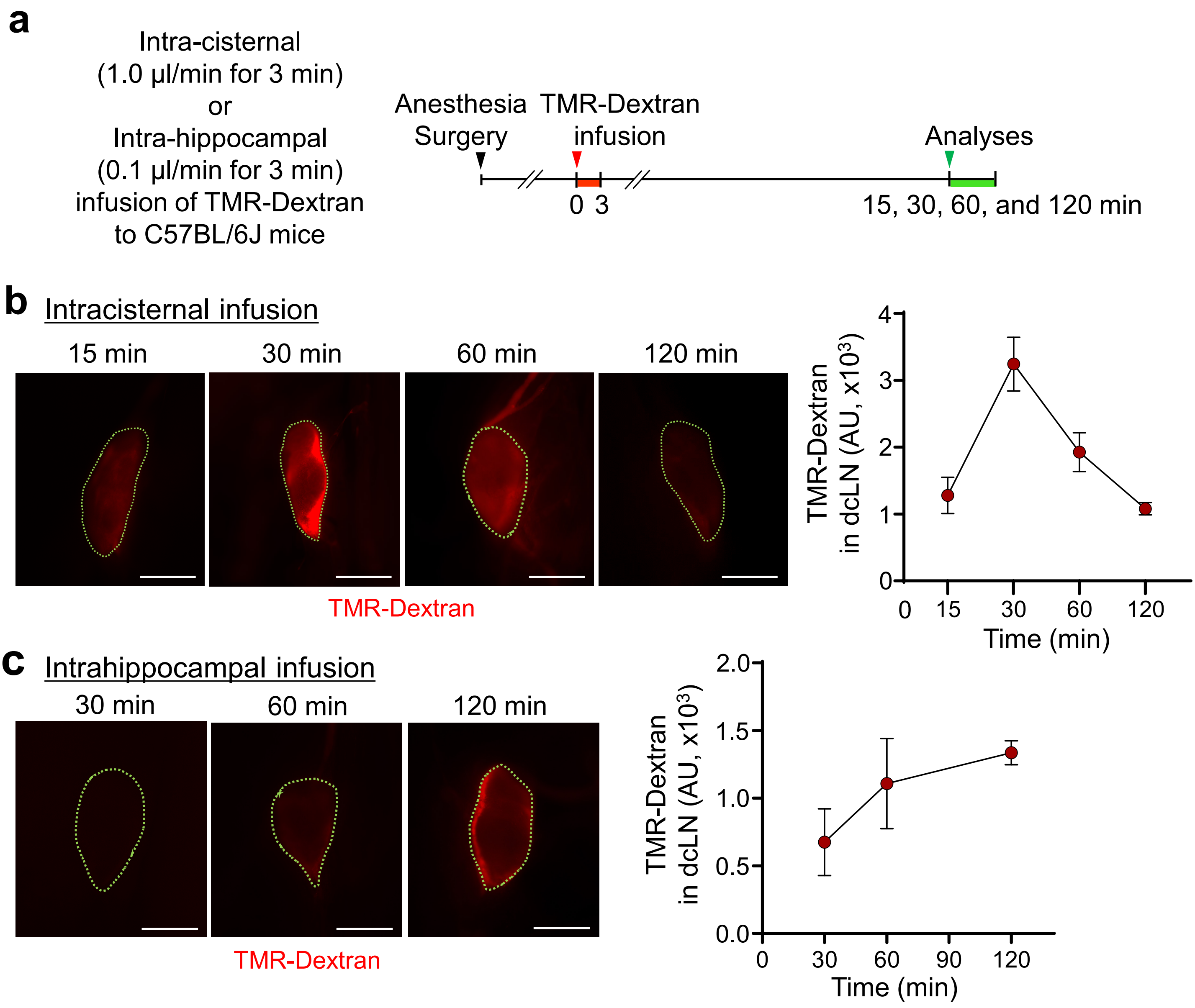

Supplementary Fig. 9. Timing of TMR-Dextran clearance to deep cervical lymph nodes after intracisternal or intrahippocampal infusion in mice

a, Diagram of experimental sequence of intracisternal or intrahippocampal infusion of TMR-Dextran at 1.0 µl/min or 0.1 µl/min for 3 min into C57BL/6J mice followed by measurement of TMR-Dextran fluorescence in deep cervical lymph nodes (dcLN) 15, 30, 60, and 120 min later.

b, Fluorescence images and measurements of TMR-Dextran fluorescence in dcLN (outlined by green dashed lines) showing peak at 30 min after intracisternal infusion. Scale bars, 500 µm. Each dot is the value for one mouse; 15 min (n=6), 30 min (n=6), 60 min (n=8), 120 min (n=6) in five independent experiments. Error bars indicate mean ± s.e.m. AU, arbitrary unit.

c, Fluorescence images and measurements of TMR-Dextran fluorescence in dcLN (outlined by green dashed lines) showing peak at 120 min after the intrahippocampal infusion. Scale bars, 500 µm. Each dot is the value for one mouse; 30 min (n=8), 60 min (n=7), 120 min (n=7) in five independent experiments. Error bars indicate mean ± s.e.m. AU, arbitrary unit.

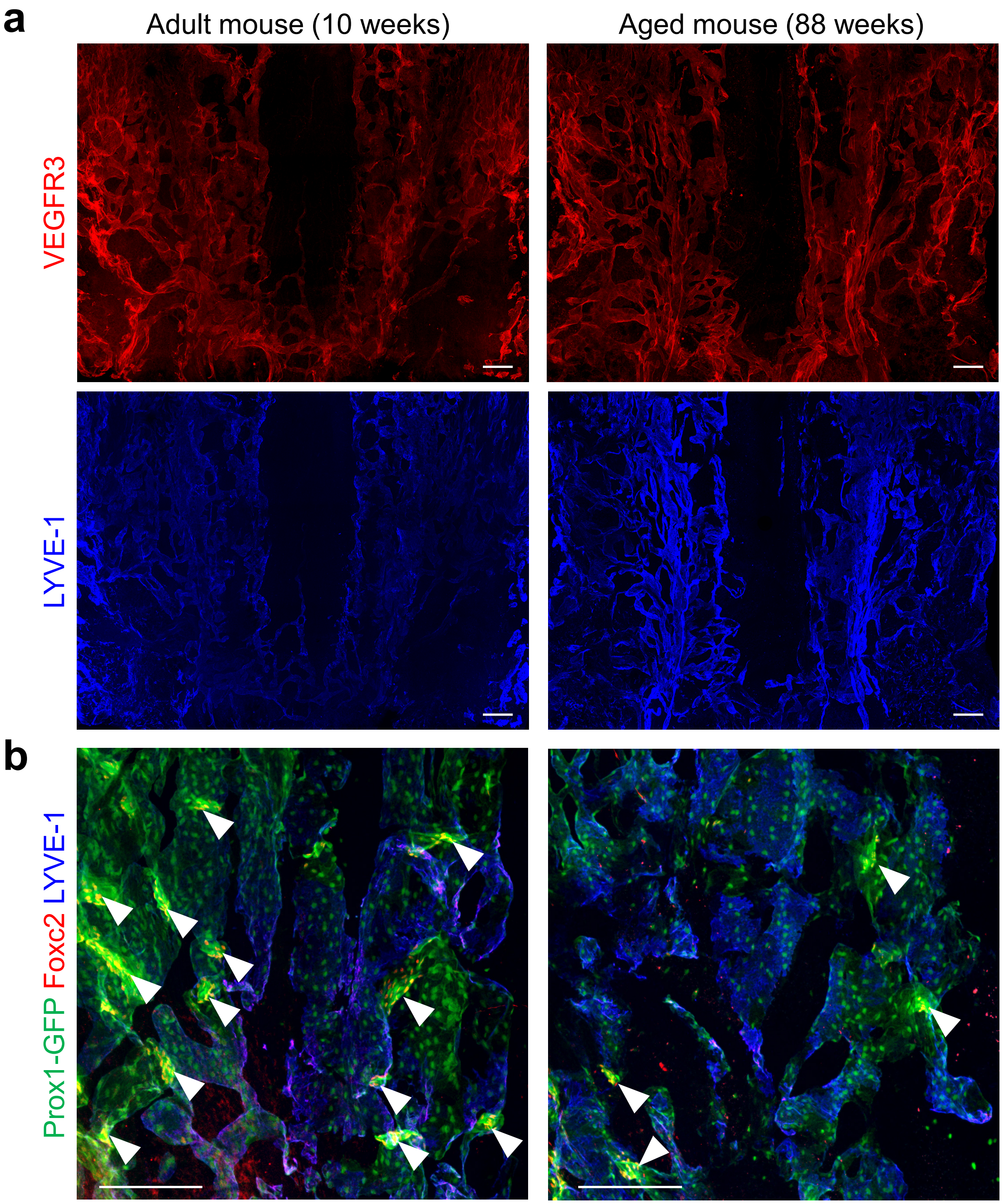

Supplementary Fig. 10. Ageing-related alterations in nasopharyngeal lymphatic plexus
a,**b**, Immunofluorescence images of whole mounts that compare two features of the dorsal region of nasopharyngeal lymphatic plexus in adult (10 weeks old) and aged (88 weeks old) mice: VEGFR3 and LYVE1 immunoreactivities in **a** and lymphatic valves stained for Prox1-GFP and Foxc2 in **b**. The regions shown are the same as in Fig. 6a, but here the images are enlarged and the color channels are separated. Regions within white dashed-line boxes are enlarged in the lower panels. Although VEGFR3 shows little change, LYVE1 increases and Prox1-dense/Foxc2^{+} valves (white arrowheads) are less numerous in the nasopharyngeal lymphatic plexus of aged mice. Scale bars, 200 μm. Similar findings were obtained from n = 4 mice in four independent experiments.

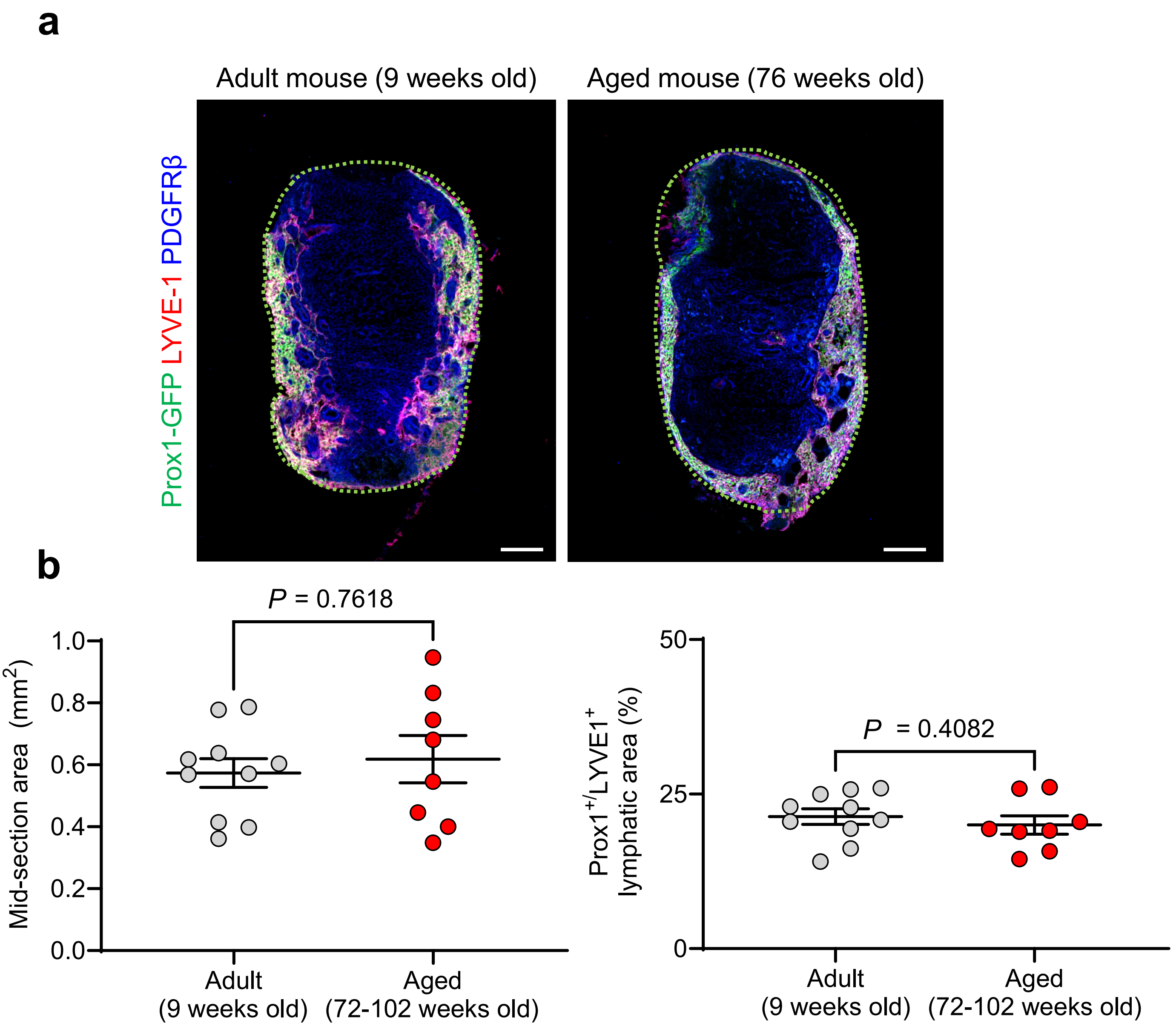

Supplementary Fig. 11. Similar size and lymphatic area of deep cervical lymph nodes in adult and aged mice

a,**b**, Immunofluorescence images comparing mid-sectioned deep cervical lymph nodes (outlined by green dashed lines) of adult (9 weeks old) and aged (73-102 weeks old) mice. Scale bars, 100 μm. Measurements show similar lymph node size and fractional area of $Prox1^{+}/LYVE1^{+}$ lymphatics. Each dot is the value for one mouse; Adult (n=8), aged (n=10) from three independent experiments. Error bars indicate mean ± s.e.m. *P* value was calculated by two-tailed Mann-Whitney test.

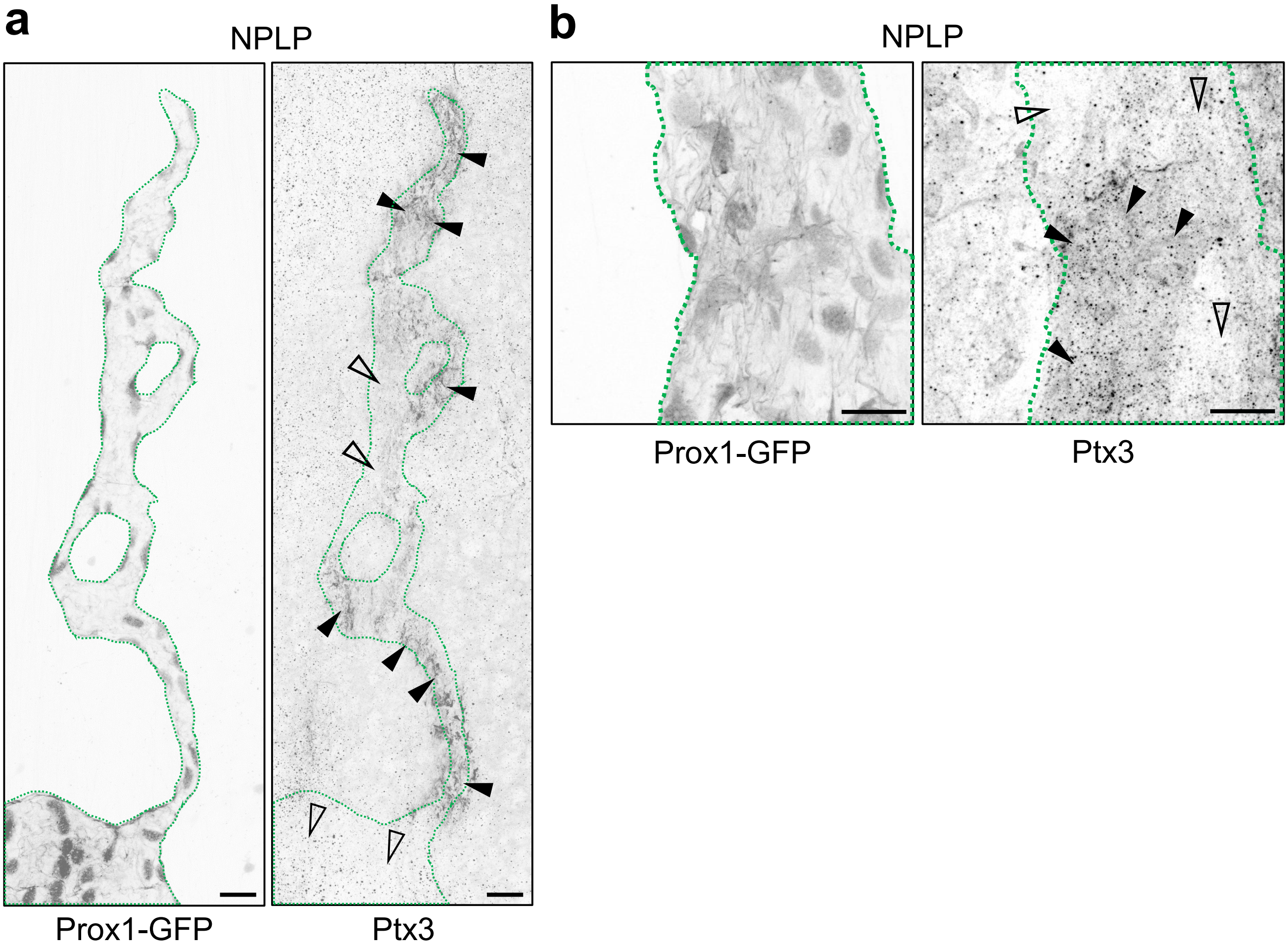

Supplementary Fig. 12. Heterogeneous expression of Ptx3 in lymphatic endothelial cells of the nasopharyngeal lymphatic plexus

a,b, Immunofluorescence images of nasopharyngeal plexus whole mounts stained for Prox1 (left) and Ptx3 (right) in Prox1-GFP mice. Immunofluorescence was converted into grayscale to highlight differences in nuclear Prox1 and extracellular Ptx3. The boundary of lymphatics, delineated by Prox1^{+} staining (outlined by green dashed lines), was superimposed onto the grayscale image of Ptx3. Ptx3 staining is diffuse in some regions and concentrated in others. **a** and **b** show two separate regions; the higher magnification of **b** illustrates diffuse Ptx3 staining. Black arrowheads mark Ptx3^{high} LECs, and open arrowheads mark Ptx3^{low} LECs. Scale bars, 20 µm. Similar findings were obtained from n = 3 mice at age 10 weeks in two independent experiments.

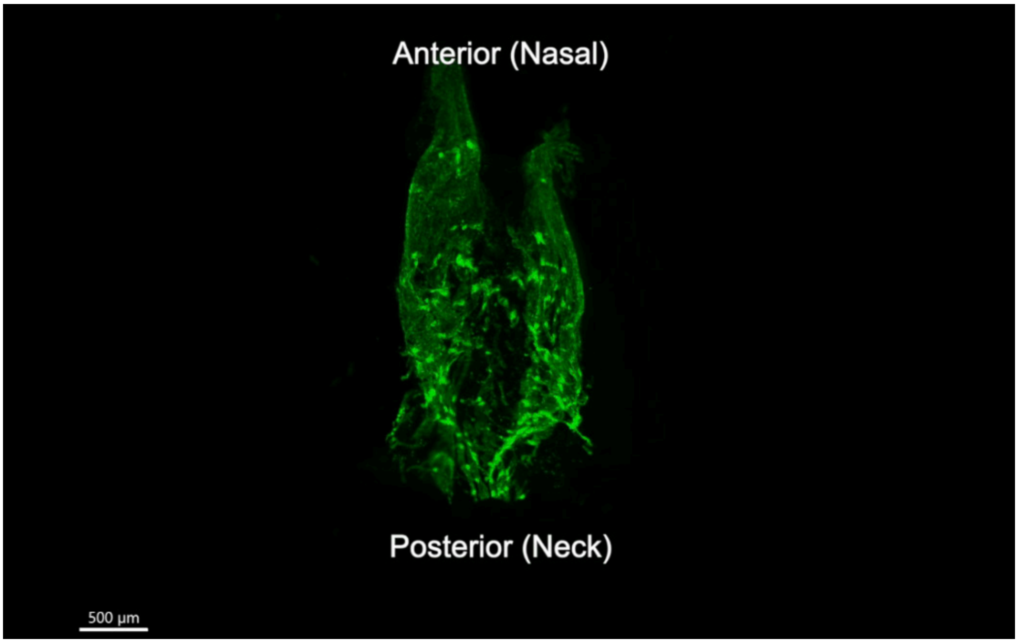

Supplementary video 1. Three-dimensional reconstruction of Prox1⁺ nasopharyngeal lymphatic plexus of Prox1-GFP mouse after tissue clearing. Scale bar, 500 µm.

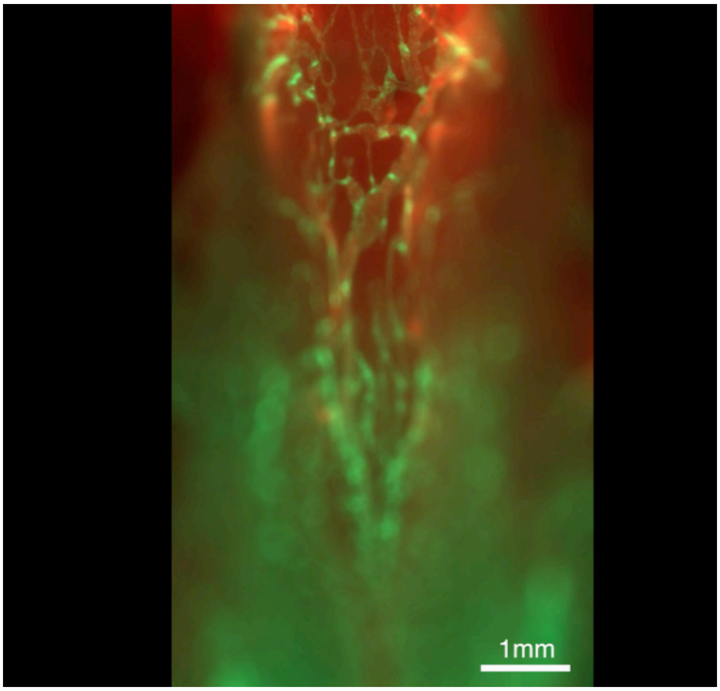

Supplementary Video 2. Intravital imaging for TMR-Dextran outflow through the nasopharyngeal lymphatic plexus at 30 min after intracisternal infusion of TMR-Dextran into Prox1-GFP mouse. Scale bar, 1 mm.

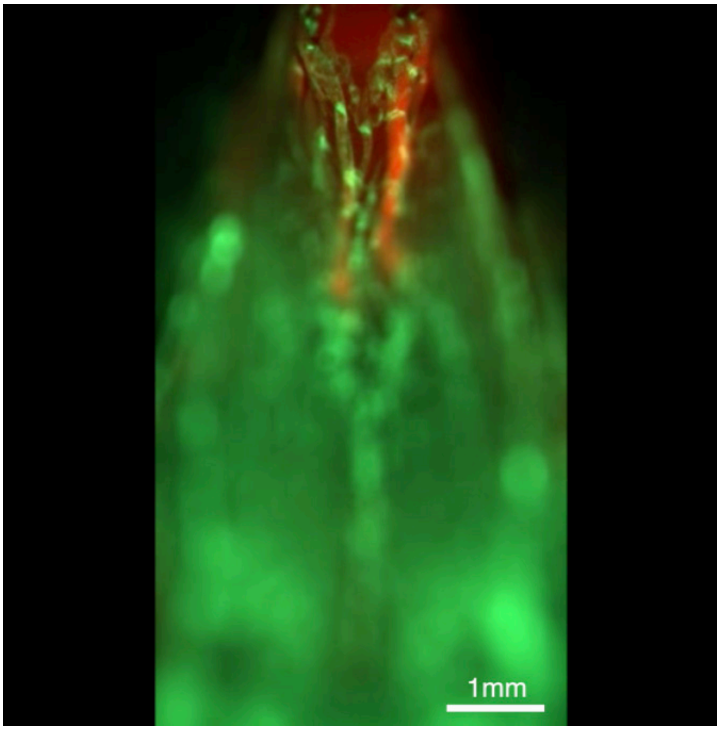

Supplementary Video 3. Intravital imaging for TMR-Dextran outflow through the nasopharyngeal lymphatic plexus at onset and 120 min after intracisternal infusion of TMR-Dextran into Prox1-GFP mouse. Scale bar, 1 mm.

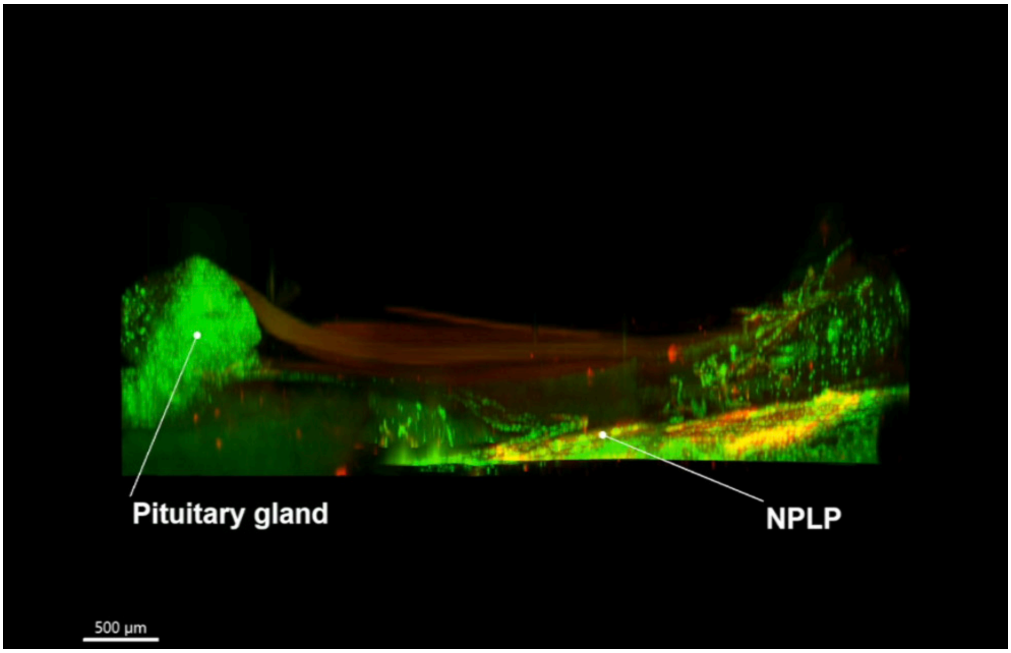

Supplementary Video 4. Light-sheet fluorescence microscopic image showing Prox1⁺/LYVE1⁺ lymphatics near the Prox1⁺ pituitary gland area that extend to the nasopharyngeal lymphatic plexus (NPLP) along cranial nerve V and cavernous sinus (white arrowhead). Scale bar, 500 µm.

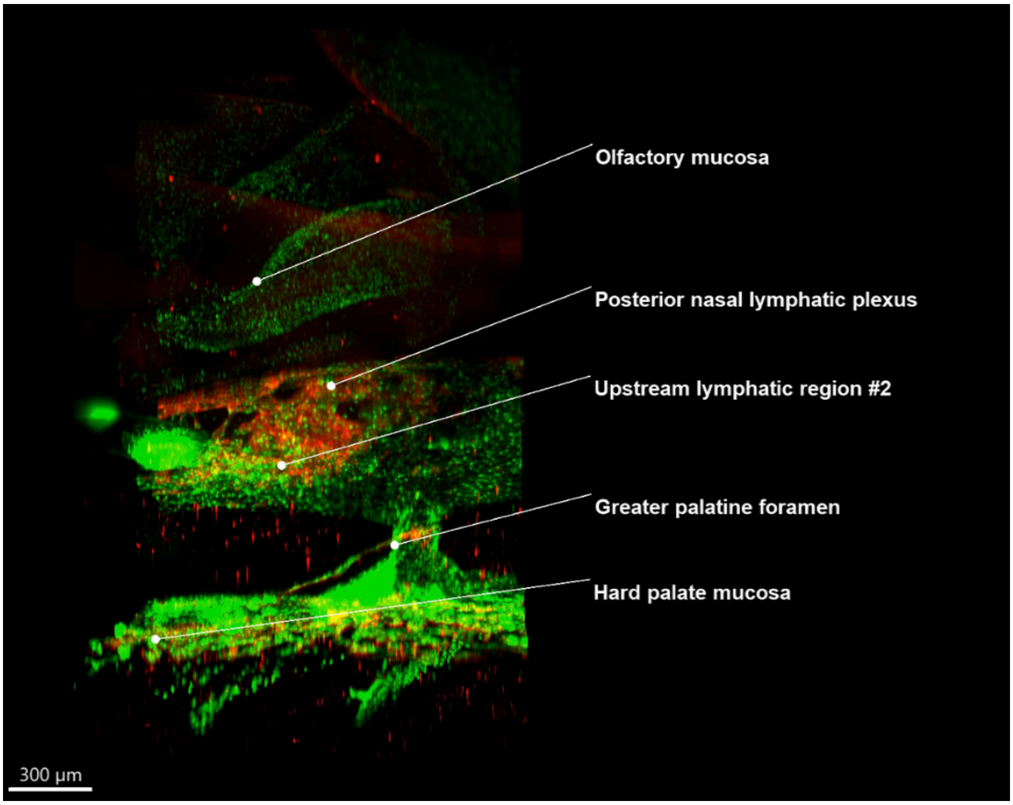

Supplementary Video 5. Light-sheet fluorescence microscopic image showing the lymphatics around the pterygopalatine artery that connect to the posterior nasal lymphatic plexus and nasopharyngeal lymphatic plexus. Scale bar, 300 µm.

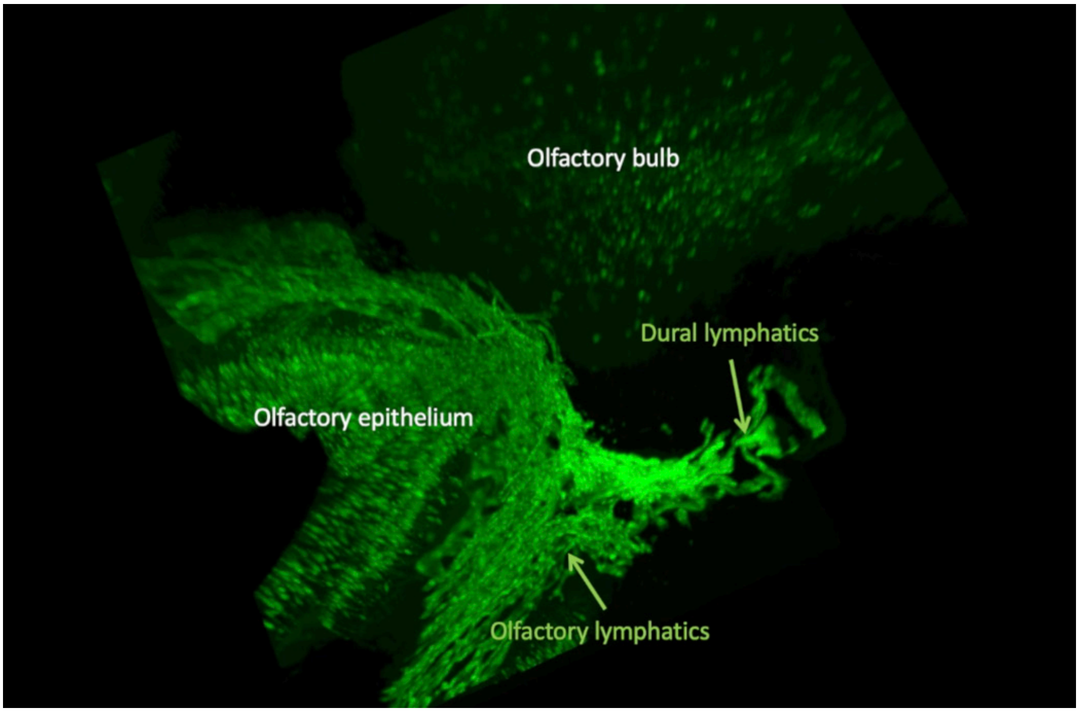

Supplementary video 6. Three-dimensional reconstruction of $Prox1^+$ connecting lymphatics between dural lymphatics and olfactory lymphatics in Prox1-GFP mouse after the tissue clearing. Scale bar, 200 μm.
