# Supplementary material for: Nasopharyngeal lymphatic plexus is a hub for cerebrospinal fluid drainage
Source: Nature. 2024 Jan 10;625(7996):768–77. doi: 10.1038/s41586-023-06899-4 (PMC10808075; doi:10.1038/s41586-023-06899-4)
Supplement: Supplementary file 2 — Reporting Summary [file 41586_2023_6899_MOESM2_ESM.pdf]

nature portfolio

Corresponding author(s): Gou Young Koh and Michael Davis

Last updated by author(s): Nov 12, 2023

Reporting Summary

Nature Portfolio wishes to improve the reproducibility of the work that we publish. This form provides structure for consistency and transparency in reporting. For further information on Nature Portfolio policies, see our Editorial Policies and the Editorial Policy Checklist.

Statistics

For all statistical analyses, confirm that the following items are present in the figure legend, table legend, main text, or Methods section.

n/a	Confirmed	
☐	☒	The exact sample size (*n*) for each experimental group/condition, given as a discrete number and unit of measurement
☐	☒	A statement on whether measurements were taken from distinct samples or whether the same sample was measured repeatedly
☐	☒	The statistical test(s) used AND whether they are one- or two-sided<br>*Only common tests should be described solely by name; describe more complex techniques in the Methods section.*
☐	☒	A description of all covariates tested
☐	☒	A description of any assumptions or corrections, such as tests of normality and adjustment for multiple comparisons
☐	☒	A full description of the statistical parameters including central tendency (e.g. means) or other basic estimates (e.g. regression coefficient) AND variation (e.g. standard deviation) or associated estimates of uncertainty (e.g. confidence intervals)
☐	☒	For null hypothesis testing, the test statistic (e.g. *F*, *t*, *r*) with confidence intervals, effect sizes, degrees of freedom and *P* value noted<br>*Give P values as exact values whenever suitable.*
☒	☐	For Bayesian analysis, information on the choice of priors and Markov chain Monte Carlo settings
☒	☐	For hierarchical and complex designs, identification of the appropriate level for tests and full reporting of outcomes
☒	☐	Estimates of effect sizes (e.g. Cohen's *d*, Pearson's *r*), indicating how they were calculated

Our web collection on statistics for biologists contains articles on many of the points above.

Software and code

Policy information about availability of computer code

Data collection	The following softwares were used for data collection:<br>LSM image software (Carl Zeiss)<br>Zen 2.3 software (Carl Zeiss)<br>Labview (National Instruments)
Data analysis	The following softwares were used for data analysis:<br>Zen 2.3 software (Carl Zeiss)<br>ImageJ software (NIH)<br>Graphpad Prism10 (GraphPad Software, version 10.1.0)<br>STAR (version 2.7.9a)<br>featureCounts (version 2.0.1)<br>R: The R Project for Statistical Computing<br>R: package 'Seurat' (version 4.1.0)<br><br>Code availability: The codes used for scRNA-seq are available at https://zenodo.org/records/10115336. The LabVIEW program used for pressure and diameter data collection of isolated lymphatic vessels is available at https://doi.org/10.5281/zenodo.8286107. The LabVIEW program used for diameter tracking of isolated lymphatic vessels is available online at https://doi.org/10.5281/zenodo.8286119.

For manuscripts utilizing custom algorithms or software that are central to the research but not yet described in published literature, software must be made available to editors and reviewers. We strongly encourage code deposition in a community repository (e.g. GitHub). See the Nature Portfolio guidelines for submitting code & software for further information.

Data

Policy information about availability of data

All manuscripts must include a data availability statement. This statement should provide the following information, where applicable:

- Accession codes, unique identifiers, or web links for publicly available datasets
- A description of any restrictions on data availability
- For clinical datasets or third party data, please ensure that the statement adheres to our policy

The scRNA-seq data of this study are available in the NCBI's Gene Expression Omnibus under accession codes GSE227311 (adult mice) and GSE227324 (aged mice). All other data supporting the findings in this study are available within the paper and its Supplementary Information. Source data are provided with this paper.

Research involving human participants, their data, or biological material

Policy information about studies with human participants or human data. See also policy information about sex, gender (identity/presentation), and sexual orientation and race, ethnicity and racism.

Reporting on sex and gender	Not applicable
Reporting on race, ethnicity, or other socially relevant groupings	Not applicable
Population characteristics	Not applicable
Recruitment	Not applicable
Ethics oversight	Not applicable

Note that full information on the approval of the study protocol must also be provided in the manuscript.

Field-specific reporting

Please select the one below that is the best fit for your research. If you are not sure, read the appropriate sections before making your selection.

☒ Life sciences ☐ Behavioural & social sciences ☐ Ecological, evolutionary & environmental sciences

For a reference copy of the document with all sections, see nature.com/documents/nr-reporting-summary-flat.pdf

Life sciences study design

All studies must disclose on these points even when the disclosure is negative.

Sample size	Sample size were chosen on the basis of standard power (with $\alpha = 0.05$ and power of 0.8) performed for similar experiments and no statistical methods were used to predetermine sample size as previously published.
Data exclusions	No samples were excluded from the analysis.
Replication	Experiments were replicated at least once for all analyses and number of reproductions of each experimental findings is described in each figure legend. All attempts at experimental replication were successful.
Randomization	Animal from different cages, but within the same experimental group, were selected to assure randomization.
Blinding	The investigators were blinded during the experiments and quantifications.

Reporting for specific materials, systems and methods

We require information from authors about some types of materials, experimental systems and methods used in many studies. Here, indicate whether each material, system or method listed is relevant to your study. If you are not sure if a list item applies to your research, read the appropriate section before selecting a response.

Materials & experimental systems

n/a	Involved in the study
☐	☒ Antibodies
☒	☐ Eukaryotic cell lines
☒	☐ Palaeontology and archaeology
☐	☒ Animals and other organisms
☒	☐ Clinical data
☒	☐ Dual use research of concern
☒	☐ Plants

Methods

n/a	Involved in the study
☒	☐ ChIP-seq
☒	☐ Flow cytometry
☒	☐ MRI-based neuroimaging

Antibodies

Antibodies used

Primary antibodies used were: anti-LYVE1 (rabbit polyclonal, 11-034, Angiobio); anti-CD31 (hamster monoclonal, clone 2H8, MAB1398Z, Merck); anti-VE-cadherin (goat polyclonal, AF1002, R&D); anti-VEGFR3 (goat polyclonal, AF743, R&D); anti-αSMA-Cy3 (mouse monoclonal, clone 1A4, C6198, Sigma); anti-β3 tubulin (mouse monoclonal, clone 2G10, ab78078, abcam); anti-Foxc2 (sheep polyclonal, AF6989, R&D); anti-LYVE1 (rabbit polyclonal, DP3500, OriGene); anti-collagen type IV (goat polyclonal, AB769, Merck); anti-Laminin α5 (rabbit polyclonal, EWL004, kerafast); anti-tyrosine hydroxylase (rabbit polyclonal, AB152, Merck), anti-vesicular acetylcholine transporter (goat polyclonal, ABN100, Merck); anti-phospho-tau (mouse monoclonal, clone AT8, MN1020, Thermo); anti-mannose receptor (CD206, rabbit polyclonal antibody, ab64693, abcam); anti-Ptx3 (rabbit polyclonal antibody, ALX-210-365-C050, Enzo Life Sciences), phycoerythrin/Cy7 anti-mouse CD326 (rat monoclonal, Ep-CAM, clone G8.8, 118216, BioLegend) antibody, APC anti-mouse podoplanin antibody (syrian hamster monoclonal, clone 8.1.1, 127410, BioLegend), and phycoerythrin-labeled anti-mouse CD31 antibody (rat monoclonal, clone MEC13.3, 102508, BioLegend). The following secondary antibodies were used: Alexa Fluor™ 488-, 594- and 647- conjugated anti-rabbit (711-545-152, 711-585-152, 711-605-152), anti-goat (705-585-147), anti-sheep (713-585-147), anti-hamster (127-605-160) secondary antibodies (Jackson ImmunoResearch). Nuclei were stained with DPAI (H-1200, Vector). Primary antibodies were diluted at 1:200 or 1:400 and secondary antibodies were diluted at 1:100 or 1:1000 for all the immunostainings or lymphatic endothelial cell sorting.

Validation

All the antibodies were validated for the species (mouse or monkey) and applications (immunohistochemistry or lymphatic endothelial cell sorting) by the correspondent manufacturer, which is described in the manufacturer's website. Our usage was described in the Methods section of the manuscript as below.

Samples were incubated in 5% normal donkey serum (017-000-121, Jackson ImmunoResearch) for 1 h at RT. Then, they were incubated with primary antibodies (1:400) dissolved in 5% normal donkey serum at 4°C for 12 h. After washing in PBS, they were incubated with secondary antibodies (1:1000) dissolved in 5% normal donkey serum at 4°C for 12 h. The samples that had been the clearing and decalcification were incubated with donkey serum for 24 h at RT, primary antibodies at 1:200 dilution at RT for 10 days, and secondary antibodies at 1:100 dilution at RT for 3 days. After PBS washing, the samples were covered with DAPI-containing mounting medium (H1200, Vector) or refractive index matching solution (D-PROTOSS).

Animals and other research organisms

Policy information about studies involving animals; ARRIVE guidelines recommended for reporting animal research, and Sex and Gender in Research

Laboratory animals

Prox1-GFP mice (8 weeks to 12 weeks old for adult, 73 weeks to 102 weeks old for aged, Choi et al., Blood, 2011; provided by Dr. Young-Kwon Hong, University of Southern California) were transferred, established, and bred in SPF animal facilities at KAIST under a 12 h-12 h light-dark cycle at 23-24 °C and 40-60 % humidity. C57BL/6J mice (8 weeks to 12 weeks old) were purchased from DBL (Soul, Korea). Aged C57BL/6J mice (73 weeks to 102 weeks old) were purchased from Animal Center of Ageing Science of Korea Basic Science Institute (Gwangju, Korea) or from JAX (USA). The head and neck portions of the primate (Macaca fascicularis, 6-13 years old) were obtained from the National Primates Center of KRIBB.

Wild animals

The study did not involve wild animals.

Reporting on sex

In this study, we randomized both genders were selected and used because gender was not a major criteria serve the purpose of this study.

Field-collected samples

The study did not involve samples collected from the field.

Ethics oversight

All animal care and experimental procedures were approved by Institutional Animal Care and Use Committees of the Korea Advanced Institute of Science and Technology (KAIST) (KA2023-014-v1) and the University of Missouri (9797) for the mice and the Korea Research Institute of Bioscience and Biotechnology (KRIBB-AEC-22237) for the primates.

Note that full information on the approval of the study protocol must also be provided in the manuscript.
